# 进展期非小细胞肺癌的首过法CT灌注可重复性研究

**DOI:** 10.3779/j.issn.1009-3419.2010.05.21

**Published:** 2010-05-20

**Authors:** 飞 单, 志勇 张, 蒙苏 曾, 洁 胡, 春学 白

**Affiliations:** 1 213003 常州，苏州大学附属第三医院影像科 Department of Radiology, the Tird Afliated Hospital of Suzhou University, Changzhou 213003, China; 2 213003 上海，复旦大学附属中山医院放射科 Department of Radiology, Zhongshan Hospital Fudan University, Shanghai 200032, China; 3 213003 上海，复旦大学附属中山医院呼吸内科 Department of Respiratory Medicine, Zhongshan Hospital Fudan University, Shanghai 200032, China

**Keywords:** 肺肿瘤, 体层摄影术, X线计算机, 灌注成像, 结果可重复性, Lung neoplasms, Tomography, X-ray computed, Perfusion imaging, Reproducibility of results

## Abstract

**背景与目的:**

本研究旨在探讨进展期非小细胞肺癌（non-small cell lung cancer, NSCLC）首过法CT灌注（CT perfusion, CTP）的可重复性。

**方法:**

在本院行首过法CTP检查（8×5 mm层厚），且经病理证实的进展期NSCLC患者14例，肿瘤最大径≤3 cm及>3 cm各7例，均在24 h内行第二次CTP扫描。采用组内相关系数（intraclass correlation coefcient, ICC）及*Bland-Altman*法评价CTP检查的可重复性。

**结果:**

两组进展期NSCLC的血流速度（blood flow, BF）、血容量（blood volume, BV）及表面通透性（permeability surface area product, PS）值的ICC均>0.75；对比剂的平均通过时间（mean transit time, MT）的ICC均 < 0.75。≤3 cm的进展期NSCLC组的BF、BV、MT及PS的可重复系数（repeatability coefficient, RC）及RC值95%变化区间依次为56%（-39%-53%）、45%（-29%-62%）、114%（-83%-145%）、78%（-57%-98%）；>3 cm组的BF、BV、MTT及PS的RC及RC值95%变化区间依次为46%（-48%-45%）、30%（-33%-26%）、-59%（-54%-64%）、33%（-18%-48%）。

**结论:**

去卷积法首过法CTP参数BF及BV可重复性较好，用于评价进展期NSCLC抗血管生成治疗疗效时，可根据肿瘤大小，应用不同的可重复性标准区别对待。

## Abstract (en)

**Background and objective:**

The aim of this study is to explore the reproducibility of volume-based quantitative measurement of non-small cell lung cancer (NSCLC) perfusion at 64-slice CT.

**Methods:**

Fourteen patients with proved advanced NSCLC were enrolled in this dynamic frst pass volume-based CT perfusion (CTP) study (8×5 mm collimation), and they underwent the second scan within 24 h. According to the longest diameters, those patients were classifed to ≤3 cm and >3 cm groups, and each group had 7 patients. Intraclass correlation coefcient (ICC) and *Bland-Altman* statistics were used to evaluate the reproducibility of CTP imaging.

**Results:**

In both groups of advanced NSCLC, the reproducibility with BF, BV, and PS values were good (ICC >0.75 for all), but mean transit time (MT) values. For advanced NSCLC (≤3 cm), repeatability coefcient (RC) values with blood flow (BF), blood volume (BV), MT and permeability surface area product (PS) values were 56%, 45%, 114%, and 78%, respectively, and the 95% change intervals of RC were -39%-53%, -29%-62%, -83%-145%, and -57%-98%, respectively. For advanced NSCLC (>3 cm), those values were 46%, 30%, 59%, and 33%, respectively, and the 95% change intervals of RC were -48%-45%, -33%-26%, -54%-64%, and -18%-48%.

**Conclusion:**

Tere is greater reproducibility of tumor size >3 cm than that of ≤3 cm. BF and BV could be addressed for reliable clinical application in antiangiogenesis therapeutic monitoring with advanced NSCLC patients.

功能成像已越来越多地用于评价肿瘤靶向治疗疗效，与MRI、PET等相比，CT灌注（CT perfusion, CTP）是临床最易获得的评价肿瘤血供的方法^[1, 2]^，在判断进展期非小细胞肺癌（non-small cell lung cancer, NSCLC）抗血管生成治疗疗效中具有重要应用前景，但其可重复性尚不明确。本研究试图探讨NSCLC的首过法CTP可重复性，为评价抗血管生成治疗疗效提供技术依据。

## 1 材料与方法

### 1.1 一般资料

本研究经复旦大学附属中山医院医学伦理委员会批准，每位患者均签署书面知情同意书。自2008年1月-2008年12月间在本院行CTP检查，且经病理或细胞学证实的Ⅲb或Ⅳ期NSCLC患者14例。选择标准：①检查前未行手术、化疗或放疗；②无对比剂过敏；③有能力配合检查；④肺内原发病灶为实性，且最大径≥1 cm；⑤CTP扫描序列无明显的图像呼吸移动。14例患者中，男性8例，女性6例，中位年龄55.5岁（34岁-72岁）；腺癌7例、鳞癌6例、肉瘤样癌1例；肿瘤最大径≤3 cm 7例（2.5 cm±0.7 cm），最大径 > 3 cm 7例（5.3 cm±1.6 cm）。

### 1.2 CTP成像

所有患者采用GE公司的LightSpeed 64机型行CTP检查。常规呼吸训练，平扫定位，以病灶最大层面为中心，行首过法同层动态增强检查，探测器宽度40 mm、5 mm×8层模式，100 kV，120 mA，曝光时间0.5 s，间隔1 s、屏气31 s、共扫描21次。用双筒高压注射器以速率5 mL/s经前臂静脉注入对比剂50 mL（300 mgI/mL），并跟随30 mL生理盐水，延迟6 s-10 s扫描，剂量长度积258.06 mGy·cm。动态增强扫描后，对比剂再次注射50 mL，速率2 mL/s，延迟25 s行全胸增强检查评价全胸其它情况。图像传入ADW 4.3工作站，采用Perfusion 3软件的体部肿瘤模式分析CTP参数，包括血流量（blood flow, BF）、血容量（blood volume, BV）、对比剂的平均通过时间（mean transit time, MTT）、表面通透性（permeability surface area product, PS）。将输入动脉感兴趣区（region of interest, ROI）设在升、降主动脉或颈总动脉。肿瘤ROI通过手动勾勒包括全瘤，并避开钙化、较大血管及病灶边缘，计算所有可测层面的平均灌注值^[3]^。14例患者在24 h内，以完全相同检查方法行第二次首过法CTP扫描^[3]^。所有图像分析由同一位放射科医生完成^[3]^。

### 1.3 统计学分析

根据肿瘤最大径，分≤3 cm及 > 3 cm两组，采用组内相关系数（intraclass correlation coefficient, ICC）及*Bland-Altman*法分析^[3, 4]^，通过MedCalc 9软件完成。ICC判断标准为数值越接近1，可重复性越好；差，ICC≤0.40；一般，0.40 < ICC≤0.74；很好，ICC > 0.75。*Bland-Altman*法分析首先通过*Kendall’s tau-b*检验评价两次灌注参数的差值与均数有无相关性，如果差值随均数增大而增大，则对两次灌注值行自然对数转换，以符合可重复性假设检验要求^[3]^。*Bland-Altman*法主要指标为两次测量值的均数、平均差值、95%一致性区间（95% limits of agreement, 95%LA）、以第二次与首次测量差值相对于首次测量值百分数的可重复性系数（repeatability coefficient，RC=1.98×两次测量差值的标准差）及RC的95%变化区间^[3]^。为与既往研究结果对比，还采用组内变异系数（within subject coefficient of variation, WCV）分析可重复性^[3, 5]^。

## 2 结果

两组NSCLC的BF、BV及PS值ICC均 > 0.75（范围0.88-0.96），提示无论肿瘤大小，ICC反映的BF、BV、PS值可重复性均很好；MTT值的ICC分别为0.30、0.59，均 < 0.75，说明可重复性差或一般，可认为其不适合判断抗血管生成治疗疗效。

经*Kendall’s tau-b*法检验，两组NSCLC两次CTP参数的差值与均数间均无相关性，*P*值均 > 0.05。BF、BV、MTT、PS值均不需经自然对数转换，可直接行*Bland-Altman*法可重复性分析。最大径≤3 cm NSCLC组各CTP参数的RC均大于 > 3 cm组的相应参数RC值。最大径≤3 cm组BF、BV、MTT、PS值的RC值95%变化区间分别为-39%-53%、-29%-62%、-83%-145%、-57%-98%； > 3 cm组的BF、BV、MTT、PS值的RC值95%变化区间分别为-48%-45%、-33%-26%、-54%-64%、-18%-48%。4个灌注参数的ICC、*Bland-Altman*法及WCV具体统计参数见表 1。

**1:** 进展期NSCLC的CTP可重复性检验（*n*=14） The reproducibility of advanced NSCLC at CTP examination (*n*=14)

CTP	Mean±SD	Mean difference^*^	WCV	RC (%)	ICC
BF (mL•min^-1^•100g^-1^)					
≤3 cm	83.5±35.8	-5.2 (-49.1-38.8)	0.21	56 (-39-53)	0.90
> 3 cm	63.9±22.0	0.5 (-27.8-28.8)	0.15	46 (-48-45)	0.89
BV (mL/100g)					
≤3 cm	4.2±1.5	0.6 (-1.1-2.3)	0.16	45 (-29-62)	0.92
> 3 cm	4.4±1.4	-0.3 (-2.3-1.7)	0.12	30 (-33-26)	0.88
MTT (s)					
≤3 cm	5.4±1.2	1.1 (-2.8-5.0)	0.20	114 (-83-145)	0.30
> 3 cm	7.2±1.6	0.4 (-3.7-4.5)	0.18	59 (-54-64)	0.59
PS (mL•min^-1^•100g^-1^)					
≤3 cm	18.0±9.1	2.4 (-7.9-12.6)	0.22	78 (-57-98)	0.92
> 3 cm	13.4±6.0	2.0 (-2.5-6.6)	0.13	33 (-18-48)	0.96
CTP: CT perfusion; WCV: within subject coefficient of variation; RC: repeatability coefficient; ICC: intraclass correlation coefficient; BF: blood flow; BV: blood volume; MTT: mean transit time; PS; permeability surface area product. ^*^Mean difference-the average of difference between two measures of CTP parameters; Data in parentheses are 95% limits of agreement.					

两组进展期NSCLC的BF及BV值的WCV、ICC及*Bland-Altman*法结果均提示可重复性良好，可被临床研究接受（图 1，图 2）。但最大径≤3 cm组PS值RC的95%变化区间较BF及BV的相应值明显大，即二次PS的差值相对首次PS值需增大98%，才可认为是药物效应所致（图 3，4）。因此，虽然≤3 cm组PS值的WCV及ICC值较好，但可重复性并不能接受。

**1:**
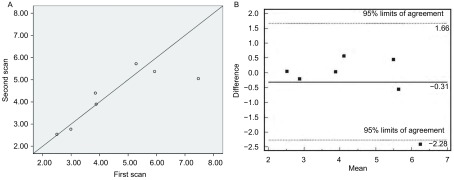
进展期NSCLC最大径 > 3 cm组的BV值散点图（A）及*Bland-Altman*图（B）。从A可见两次参数值在对角线两侧分布较均匀，仅一个点偏离较远。从B可见7例患者中有1例位于95%LA外，界限内变异最大的点约0.6 mL/100g，相对均值4.4 mL/100g，可重复性可接受。 Scatter plot (A) and *Bland-Altman* agreement plot (B) of difference between the two studies against mean of BV median values from the two studies of measurements in patients with advanced NSCLC (the group of tumor diameter > 3 cm). The closer the plots lie to this line of equality, the better the reproducibility, and only one plot is far away from the diagonal (A). Mean difference is indicated by solid line. Two outer dotted lines represent 95%LA, which define range within which most differences between repeated BV measurements made on the same subject will lie. There was an obvious outlier, and the most variant difference is 0.6 mL/100g, related to average of 4.4 mL/100g in the line of 95%LA, so the reproducibility can be acceptable (B).

**2:**
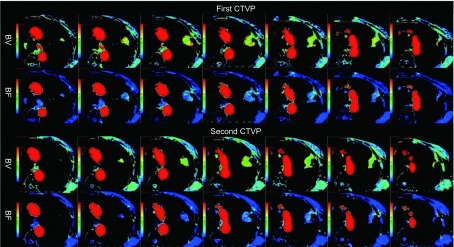
男性，62岁，左肺上叶鳞癌，Ⅲb期，最大径约3.5 cm。CTP检查获得的参数值变化较小：第一次BV=5.93 mL/100g，BF=84.11 mL•min^-1^•100g^-1^；第二次BV=5.37 mL/100g，BF=84.03 mL•min^-1^•100g^-1^。 A male, 62 years old, stage IIIb, had a squamous cell carcinoma in the left upper lobe, and the diameter was 3.5 cm. The variations of CTP examination were small: BV value was 5.93 mL/100g and BF value was 84.11 mL•min^-1^•100g^-1^ in the first scan; BV value was 5.37 mL/100g and BF value was 84.03 mL•min^-1^•100g^-1^ in the second scan.

**3:**
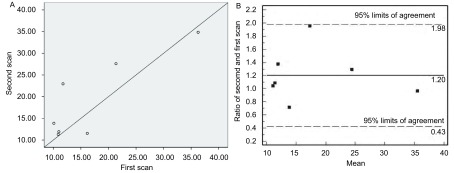
进展期NSCLC最大径≤3 cm组PS值的散点图（A）及*Bland-Altman*图（B）。从A散点图可见两次参数值在对角线两侧分布较分散。该*Bland-Altman*图纵坐标为PS第二次值相对于首次值的比率，95%变化范围为0.43-1.98，即第二次相对于首次PS值变化的95%范围在减低57%至增大98%之间；超过这个区间才能认为两次PS值不同；界限内变异最大的点对应于第二次较首次增大了98%，对判断抗血管生成疗效而言，可重复性不可接受。 Scatter plot (A) and *Bland-Altman* agreement plot (B) of ratio of the second scan and first scan between the two studies against mean of PS median values from the two studies of measurements in patients with advanced NSCLC (the group of tumor diameter ≤3 cm). The points representing two measurements lie dispersively between two sides of the this line of equality (A). The 95%LA is from 0.43 to 1.98, just means the 95% variation limits of the second PS value is between -57% decreasingly and 98% increasingly, compared to the first scan. The second measurement can be thought different, only if which exceed the 95% LA interval, and the most variant point in the interval is 98% increasingly, so for antiangiogesis therapeutic assessment, the repeatability can not be acceptable (B).

**4:**
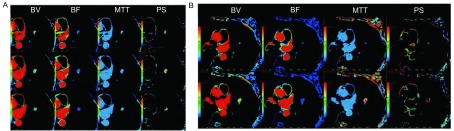
女性，34岁，左肺上叶腺癌，Ⅳ期，最大径约2.1 cm。患者第二次检查时，定位扫描与动态增强扫描的屏气程度不一，致扫描未完全覆盖肿瘤，CTP参数值变化较大，第二次PS值较首次增大了95%，MTT增大了153%，BV、BF值也有较大变化，分别增大了28%及降低了27%。A为第一次CTP，BV=3.46 mL/100g，BF=87.47 mL•min^-1^•100g^-1^，MTT=3.11 s，PS=11.75 mL•min^-1^•100g^-1^；B为第二次CTP，BV=4.44 mL/100g，BF=64.10 mL•min^-1^•100g-^-1^，MTT=7.38 s，PS=22.95 mL•min^-1^•100g^-1^。 A female, 34 years old, stage Ⅳ, had a adenocarcinoma in the left upper lobe, and the diameter was 2.1 cm. When the second examination was performed, CTP didn't cover the whole tumor, owing to the different breath holding. The CTP parameters varied much: the second PS value was 95% increasingly, the MTT value was 153% increasingly, the BV value was 28% increasingly, and the BF value decreasingly 27%, compared with the first CT scan. In the first CTP, the BV value was 3.46 mL/100g, the BF value was 87.47 mL•min^-1^•100g^-1^, the MTT value was 3.11 s, and the PS value was 11.75 mL•min^-1^•100g^-1^ (A). In the second CTP, the BV value was 4.44 mL/100g, the BF value was 64.10 mL•min^-1^•100g^-1^, the MTT value was 7.38 s, and the PS value was 22.95 mL•min^-1^•100g^-1^ (B).

## 3 讨论

抗血管生成疗法已成为治疗晚期肿瘤的新希望，但疗效却可不表现为肿瘤退缩，故传统以肿瘤大小为基础的疗效评价标准受到了质疑^[5]^。研究^[6]^认为通过RECIST标准测定疗效，可低估该类药物治疗的生物学活性。因CTP可无创性评价肿瘤血管生成、MSCT软硬件的不断发展，使通过CTP评价抗血管生成类药物治疗晚期肿瘤疗效成为研究热点。CT对比剂的药物动力学特性表现为碘浓度与CT值高度线性相关，故基于CT动态增强的CTP值应具有高精确性^[5]^。然而，实际计算受多因素影响，结果并不那么理想。

### 3.1 NSCLC的CTP测量误差和影响可重复性的主要因素

NSCLC的CTP固有测量误差主要来源于两种：第一种为内源性误差：包括软件计算误差、肿瘤血流灌注的异质性、患者的心输出量和屏气状态；第二种为外源性误差：如观察者差异^[3, 5]^。这两种误差相对治疗前后CTP差异应足够小，才能减少疗效误判。

具体而言，肺癌CTP测量变异主要来源于以下几点。首先是设定输入动脉兴趣区（region of interest, ROI）^[5]^。CTP软件计算要求选择输入动脉，体部临床应用时一般选择主动脉代替肿瘤供血动脉。受到对比剂注射及患者CT检查时不同心输出量影响，CTP输入动脉时间-密度曲线（time density curve, TDC）的细微差别，尤其是上升段会影响去卷积法数学计算过程，进而影响计算BF及MTT，最后影响测量值的可重复性^[7]^。

其次是部分容积效应。胸部CTP检查时，长时间屏气造成输入动脉及肿块不可避免的呼吸移动。每次检查时，患者不等的呼吸移动引起CT层面内不同的容积效应，再加上瘤-肺界面由CT值差异极大的肿瘤实质与瘤周空气构成影响，虽然层厚采用5 mm，但却不可能完全消除输入动脉、肿瘤实质及瘤-肺界面的部分容积效应。肿瘤范围ROI也是重要因素^[5]^。实际上，即使同一位医生，因瘤-肺界面不光整及容积灌注测量多个CT层面，手动勾勒多次ROI，会影响到可重复性。

CTP软件的数学模型也会对可重复性造成重要影响^[5]^。Goh等^[8]^研究提示不同数学模型计算出的CTP参数值有较大变异。所以使用不同CTP软件、不同机型的研究结果具有独立性，不能相互比较，以保证灌注检查的可重复性。本研究采用的去卷积软件分析通过测量对比剂注射后的MTT完成，脉动剩余函数（impulse residue function, IRF）利用已知的输入动脉和靶组织TDC计算。利用IRF的TDC可计算出灌注参数。但IRF不是一个常量，受到潜在噪声影响，虽然去卷积模型通过引入常数项来进行校正，但即使在起始数据、输入动脉及靶组织ROI、观察者一致的情况下，每次测量值也不一样，仍有13.2%的变化^[5]^。Ng等^[3]^的研究也说明数学模型对灌注参数的影响大。

### 3.2 分析可重复性的统计方法

以往研究^[5]^多采用简单相关或回归来分析可重复性。但随后发现运用简单相关或回归评价可重复性是不正确的^[4, 5, 9]^，易受到极限值影响，高相关性的两组数可重复性可能并不好^[5]^。而*Bland-Altman*法分析对样本量要求小、对极限值不敏感，结果更加稳定^[4, 5]^。ICC虽然比简单相关分析更适合于评估可重复性，但高相关性实际上可能并不足以满足临床医疗实践要求的高一致性，必须结合*Bland-Altman*法的95%LA具体判断^[3, 5]^。*Bland-Altman*法较其它统计学方法的优点还在于可定量两次测量值的变化范围^[4]^。RC值的95%变化区间可认为是判断抗血管生成治疗晚期肿瘤疗效时，前后两次CTP无变化的范围^[3]^。

### 3.3 进展期NSCLC的首过法CTP可重复性

已知抗血管生成类药物治疗晚期肿瘤后的血流灌注变化在30%-90%之间，而多数CTP研究的变异系数在15%-30%间。本研究进展期NSCLC的4个CTP参数的WCV值为7%-29%，与Ng等^[3]^的结果相似，理论上均可运用于监测疗效。但我们进一步通过ICC及*Bland-Altman*法分析发现，只有参数BF及BV的可重复性适合判断抗血管生成治疗疗效的需要。

对比最大径≤3 cm和 > 3 cm两组NSCLC的BF及BV值可重复性分析结果，虽然WCV及ICC值相差不大，但*Bland-Altman*法分析发现前者的RC及95%变化区间均大于后者，说明≤3 cm组NSCLC的CTP可重复性小于 > 3 cm组。这种差异的原因可能是肿瘤血流灌注异质性所致：两次CTP检查时，因Z轴覆盖范围为4 cm，直径小的NSCLC会因患者肿瘤定位和动态增强扫描的屏气程度差异，丢失部分肿瘤区域，对容积灌注计算产生较大影响；直径较大的肿瘤则因CT扫描仍可覆盖肿瘤较多部分，故计算出的容积灌注值变异较小。这一现象提示在判断抗血管生成药物疗效时，如不能实现全瘤灌注，应将NSCLC按肿瘤大小运用不同的可重复性标准区别对待。

本研究的不足之处有以下3点：首先，CT动态增强未延迟至对比剂注射后2 min以上，仅采用首过法扫描。这样虽可避免长屏气引起图像呼吸移动及CTP错误计算，但所获得的PS值不足以反映肿瘤微血管渗透性^[10, 11]^。其次，受到Z轴覆盖4 cm范围的限制，本研究无法实现全部病灶的全瘤CT灌注。采用“shuttle”模式CTP或配备更宽探测器的MSCT是解决这一问题的方向。再次，CTP检查的X线辐射剂量高，首过法动态增强的剂量长度积达到258.06 mGy·cm。但相对于晚期恶性肿瘤患者的放射治疗，仍是可以接受的^[11]^。

总之，去卷积法首过法CTP参数BF及BV可重复性较好。用于评价进展期NSCLC抗血管生成治疗疗效时，应注意根据肿瘤大小，选择不同的可重复性标准区别对待。
